# *Staphylococcus aureus* biofilm elicits the expansion, activation and polarization of myeloid-derived suppressor cells *in vivo* and *in vitro*

**DOI:** 10.1371/journal.pone.0183271

**Published:** 2017-08-16

**Authors:** Kuo-Ti Peng, Ching-Chuan Hsieh, Tsung-Yu Huang, Pei-Chun Chen, Hsin-Nung Shih, Mel S. Lee, Pey-Jium Chang

**Affiliations:** 1 Department of Orthopedic Surgery, Chang Gung Memorial Hospital, Chiayi, Taiwan; 2 Department of Surgery, Chang Gung Memorial Hospital, Chiayi, Taiwan; 3 Division of Infection Disease, Department of Internal Medicine, Chang Gung Memorial Hospital, Chiayi, Taiwan; 4 Graduate Institute of Clinical Medical Sciences, College of Medicine, Chang Gung University, Taoyuan, Taiwan; 5 Department of Orthopedic Surgery, Chang Gung Memorial Hospital, Taoyuan, Taiwan; 6 Department of Orthopedic Surgery, Chang Gung Memorial Hospital, Kaohsiung, Taiwan; 7 Department of Nephrology, Chang-Gung Memorial Hospital, Chiayi, Taiwan; Universidad de Palermo, UNITED STATES

## Abstract

*Staphylococcus aureus* (*S*. *aureus*) is one of the most common causes of biofilm infections in periprosthetic joint infections (PJIs). Accumulating evidence has shown that the immunosuppressive environment established by *S*. *aureus* biofilm infection in PJIs involves the presence of myeloid-derived suppressor cells (MDSCs) and M2-macrophages. Due to the diversity of MDSCs, little is known about whether *S*. *aureus* biofilm preferentially expands specific MDSC subsets or whether MDSCs can further differentiate into M2-macrophages during *S*. *aureus* biofilm infection. Here, we show that in agreement with the results from an established rat PJI model, *S*. *aureus* biofilm cocultured with freshly isolated bone marrow cells (BMCs) *in vitro* significantly increases the proportions of MDSCs, total macrophages and M2-macrophages. Interestingly, we find that treatment of the BMCs *in vitro* with *S*. *aureus* biofilm preferentially promotes the expansion of monocytic MDSCs but not granulocytic MDSCs. Biofilm treatment also substantially enhances the overall MDSC immunosuppressive activity in addition to the MDSC expansion *in vitro*. Importantly, we provide evidence that *S*. *aureus* biofilm is capable of further stimulating the conversion of monocytic MDSCs into M2-macrophages *in vitro* and *in vivo*. Collectively, our studies reveal a direct link between MDSCs and M2-macrophages occurring in *S*. *aureus*-associated PJIs.

## Introduction

Prosthetic joint infections (PJIs) are a devastating complication after arthroplasty, which greatly affect the quality of a patient’s recovery [1–3]. The incidence of PJIs is about 1% to 2% for primary total joint arthroplasties, and approximately at 2%—6% for a revision joint arthroplasty [4]. Treatment strategies include radical debridement with retention of the implants, one-stage (or immediate) revision, two-stage (or delayed) revision, and excision arthroplasty [5]. However, the reinfection rate after re-implantation of the prosthesis is still high and more than 10% in the follow up [6, 7].

*Staphylococcus aureus* (*S*. *aureus*), a Gram-positive microorganism, is a major human pathogen and a major cause of community-associated and nosocomial infections. It also elicits biofilm infection on orthopedic implants or other internal medical devices. Biofilms are surface-associated communities of bacteria enclosed within a self-produced matrix, which is consisted of mainly polysaccharides, nucleic acids, proteins and lipids. *S*. *aureus* biofilm infection in PJIs often causes a serious health care concern based on their protection from antibiotic treatment and the host’s immune system. Currently, the methicillin-resistant *S*. *aureus* (MRSA) has gradually increased its presence in the human population causing this pathogen as a greater therapeutic problem [8]. The development of vaccines or immunotherapies to treat *S*. *aureus*-associated biofilm infection is an urgent need in the realm of public health [9, 10].

Myeloid-derived suppressor cells (MDSCs) are a heterogeneous population of activated immature myeloid cells with the ability to suppress T cell responses. Intrinsically, MDSCs can be discriminated from mature myeloid cells (i.e., dentritic cells, macrophages, and neutrophils) by unique cell-surface marker signatures. For example, MDSCs in mice are commonly characterized as CD11b^+^Gr1^+^ cells, whereas MDSCs in rats are identified as CD11bc^+^His48^+^ cells. Due to the heterogeneity of these immature myeloid cells, MDSCs can be further classified into two subsets: monocytic MDSCs (M-MDSCs) and granulocytic MDSCs (G-MDSCs). Morphologically, M-MDSCs are immature mononuclear cells similar to monocytes, while G-MDSCs are immature polymorphonuclear cells similar to neutrophils. In mice, the Gr1 marker is composed of two components: Ly6G and Ly6C, which can serve as the subset markers for M-MDSCs (CD11b^+^Ly6C^high^Ly6G^-^) and G-MDSCs (CD11b^+^Ly6C^low^Ly6G^+^) [11]. In clinical investigations, MDSCs have been shown to accumulate during pathological situations, such as tumor development, acute or chronic inflammation and trauma, as well as bacterial infection [12, 13]. Although several studies have previously shown that MDSC expansion could occur during *S*. *aureus* biofilm infection [14], little is known about whether there is a preferential expansion of specific subsets within MDSCs in response to *S*. *aureus* biofilm.

In addition to MDSC expansion, *S*. *aureus* biofilm also exhibits the ability to polarize macrophages toward the alternatively activated (M2) macrophages [15], which are distinct from the classically activated (M1) macrophages [16]. M1-macrophages are considered as pro-inflammatory macrophages involved in host defense against bacterial infections, whereas M2-macrophages are responsible for anti-inflammation and bacterial persistence [17–23]. M2-macrophages also function to suppress T cell proliferation [24]. Activation of M2-macrophages is usually associated with abundant expression and secretion of anti-inflammatory factors such as Arginase-1, IL-10 and IL-12, and thereby restrains a pro-inflammatory immune response [25, 26]. Although both MDSCs and M2-macrophages play critical roles in the immune suppression during *S*. *aureus* biofilm infection, it remains unclear if MDSCs could further differentiate into M2-macrophages upon exposure to *S*. *aureus* biofilm.

In this report, both *in vivo* and *in vitro* model systems were designed to examine the production of both MDSCs and M2-macrophages during *S*. *aureus* biofilm stimulation. In a rat PJI model, we did find that circulating MDSCs and M2-macrophages were accumulated in the peripheral blood during *S*. *aureus* biofilm infection. Coculture of bone marrow precursor cells (BMCs) with *S*. *aureus* biofilm *in vitro* also caused a significant increase in the proportions of MDSCs and M2-macrophages. Specially, M-MDSCs, but not G-MDSCs, were preferentially expanded in the *in vitro* coculture system. Moreover, results from both *in vitro* and *in vivo* studies supported that MDSCs could turn into M2-macrophages in the presence of *S*. *aureus* biofilm. A detailed understanding of the development of MDSCs and M2-macrophages during *S*. *aureus* biofilm infection may allow us to develop better strategies in the treatment of *S*. *aureus*-associated PJIs or chronic osteomyelitis.

## Materials and methods

### Rats and mice

Male LEW/SsNNarl rats (12 weeks of age) with 350–400 gram and male C57BL/6J mice (12 weeks of age) with 22–25 gram obtained from National Laboratory Animal Center (Taiwan) were used for *S*. *aureus* biofilm infection. The Pgk1-EGFP transgenic mice (C57BL/6J-Tg[Pgk1-EGFP]03Narl) (National Laboratory Animal Center, Taiwan) were utilized to provide the CD11b-positive MDSCs that express EGFP. All animal experiments were approved by the Institutional Animal Care and Use Committee of the Chang Gung Memorial Hospital (IACUC permit number: 2011102502, 2012122508 and 2013122701), and were performed in accordance with the Animal Protection Law by the Council of Agriculture, Executive Yuan (R.O.C.) and the guideline of National Research Council (U.S.A) for the care and use of laboratory animals.

### *S*. *aureus* strain and biofilm formation

*S*. *aureus* (ATCC43300) was purchased from Bioresource Collection and Research Center (BCRC), Taiwan. To prepare *S*. *aureus* biofilm, the bacteria were cultured in BHI media for 4 days as previously described [15, 27]. Biofilm communities were collected by centrifugation at 3900 xg for 15 min, and then sterilized by autoclaving. Biofilm pellets were resuspended with RPMI medium and total protein amounts of the lysates were measured using BCA protein assay kit (Pierce). For the *in vivo* study, *S*. *aureus* cells were pelleted, washed 3 times, and resuspended in normal saline. Bacterial concentrations were estimated spectrophotometrically by measuring the absorbance at 600 nm (A_600_). Colony forming units (CFU) were counted on the plates to verify the optical measurement.

### *S*. *aureus* biofilm infection in rats and in mice

The rat model of the *S*. *aureus* orthopedic implant infection was performed as described previously with minor modifications [28]. Briefly, male LEW/SsNNarl rats were anesthetized with a 1:1 mixture of Zoletil 50 and Rompun 2% (1 ml/kg in rat and mice) and the surgical site was disinfected with povidone-iodine. A skin incision was made near the right knee. A burr hole was made in the femoral intercondylar notch extending into the intramedullary canal using a 26-gauge needle, and then a pre-cut 0.5 cm orthopedic grade Kirschner (K)-wire (0.6-mm diameter, Synthes GmbH, Switzerland) was implanted into the intramedullary canal. Following the insertion of K-wire implant, 2×10^3^ CFU of *S*. *aureus* were inoculated. Subsequently, the burr hole was sealed with bone wax and then the surgical site was closed with Coated VICRYL* 4–0 sutures. Rats that received sterile implants served as the sham-operated group. For pain relief, rats were treated with Ketorolac (0.5~1 mg/kg, IM) immediately after the surgery and 24 hours later. For the PJI model in mice, all the procedures of *S*. *aureus* infection were the same as those in rats. Herein, after infection with *S*. *aureus* for 7 days in C57BL/6J mice, 1×10^7^ CD11b-positive MDSCs isolated from Pgk1-EGFP transgenic mice were injected into the infected mice intravenously. Twenty-four hours later, the mice were sacrificed and the tissue near the *S*. *aureus* infected site were taken, embedded and frozen in -80°C.

### Scanning electron microscopy

Rats were sacrificed at day 7 after *S*. *aureus* infection. The bone rougeur was used to cut the femur longitudinally and a piece of fragment (2×4 mm^2^) was picked. Femur samples were fixed in 0.1M cacodylate buffer (pH 7.4) containing 3% glutaraldehyde and 2% paraformaldehyde for 2 hr at 4°C. Subsequently, fixed specimens were cut longitudinally and washed three times in 0.1 M cacodylate buffer (pH 7.4) for 10 minutes at 4°C. In the fume hood, specimens were soaked in 0.1 M cacodylate buffer containing 1% Osmium Teteroxide for 1 hr at 4°C. After rinsing three times using 0.1M cacodylate buffer for 10 min at 4°C, specimens were dehydrated using a graded series of ethanol washes and critical-pointed-dried using a critical point dryer (CPD). Dried specimens were coated with gold-palladium. Samples were viewed using a HITACHI S-5000 scanning electron microscope.

### Flow cytometry analysis

To measure the dynamic changes in the proportions of immune cell populations in the peripheral blood of rats, 50 μl of blood samples was taken for flow cytometry analysis. After lysing red blood cells, the remaining leukocytes were resuspended in PBS containing 2% FBS, and then were stained with HIS48-FITC (eBioscience), CD11bc-allophycocyanin (BioLegend), CD68-PE (Biolegend) or CD206-PerCP (Abcam) for 30 min at 4°C. On the other hand, mice cells were analyzed by staining with CD11b-FITC (BD Bioscience), Gr-1-PE, Ly6G-APC (BD Bioscience), Ly6C-PerCP-Cy5.5 (eBioscience), F4/80-PE (eBioscience) or CD206-PerCP (Biolegend, #141716). Acquisition of flow cytometry data from the BD FACSCantoII flow cytometer was performed using FACSDiva software (BD Biosciences). The number of events analyzed was 10,000 per sample. Analysis was performed using FlowJo software (Tree Star).

### Isolation of specific immune cell populations from mice bone marrow

BMCs were collected from bone marrow of mouse femur. The CD11b-positive cell population was isolated using anti-CD11b antibody conjugating with biotin in combination along with anti-biotin magnetic beads (STEMCELL^™^) according to the manufacturer’s instructions. The purity of the isolated cell population was verified by flow cytometry. Two MDSC subsets, M-MDSCs (Ly6C^high^Ly6G^-^) and G-MDSCs (Ly6C^low^Ly6G^+^), were then sorted from the CD11b-positive population by using BD FACSAria Fusion cell sorter.

### Coculture of the isolated cell populations with *S*. *aureus* biofilm *in vitro*

Mouse BMCs or the isolated cell populations were seeded in the 24-well plate and cocultured with different concentrations of *S*. *aureus* biofilm loaded in the transwell cell culture insert (0.4 μm pore size). All the cocultures of the indicated cell populations with *S*. *aureus* biofilm were maintained in complete medium (RPMI1640 with 10% FBS). After coculture for 48 or 72 hr, the biofilm-treated cells were analyzed by flow cytometry (BD FACSCanto II) using specific antibodies.

### T cell proliferation assays

Mouse BMCs were seeded in 24-well plates and then left untreated or treated with *S*. *aureus* biofilm for 48 hr. After 48-hr treatment, the CD11b-positive cell population from the untreated or biofilm-treated BMCs was isolated using anti-CD11b antibody conjugating with biotin in combination along with anti-biotin magnetic beads (STEMCELL^™^). The untreated and biofilm-treated BMCs as well as their isolated CD11b-positive cell populations were subsequently cocultured with CFSE-labeled spleen T cells that were stimulated by Dynabeads Mouse T-Activator CD3/CD28 (Gibco). Different ratios of BMCs (or the isolated CD11b-positive cells) to activated T cells (2×10^5^ cells/well in a 96-well round bottom plate in RPMI-1640 with 10% FBS) were examined in the study.

For isolation of spleen T cells, mouse spleens were gently pressed through a strainer using the plunger end of a syringe to get singlet cell. These cells were washed with PBS buffer and ACK lysing buffer (Gibco). Cells in warm RPMI (2 ml) were injected to nylon wool column, and washed with RPMI. The column was sealed and incubated at 37°C and 5% CO2 for 45 minutes. Cells were then eluted with 10 ml of warm RPMI.

### Analysis of regulatory T (Treg) cells

Mouse BMCs were seeded in 24-well plates and then left untreated or treated with *S*. *aureus* biofilm. After 48-hr treatment, the CD11b-positive cell populations were isolated from the untreated or biofilm-treated BMCs. The isolated CD11b-positive cell populations were then cocultured with spleen T cells that were stimulated by Dynabeads Mouse T-Activator CD3/CD28 (Gibco). After 24- or 48-hr coculture, the proportions of Foxp3^+^CD25^+^CD4^+^ Tregs in the cell mixtures were analyzed by flow cytometry using a True-Nuclear One Step Staining Mouse Treg Flow kit (FOXP3 Alexa Fluor 488/CD25 PE/CD4 PerCP; Biolegend). Results were expressed as the percentages of Foxp3^+^CD25^+^CD4^+^ T cells in CD4^+^ cells.

### Quantitative reverse transcription (RT)-PCR

Total RNAs were isolated from cells using an RNeasy Mini Kit (Qiagen, Valencia, CA) in combination with an RNase-free DNase set (Qiagen, Valencia, CA). RNA samples were first converted into cDNAs using RevertAid First Strand cDNA Synthesis Kit (Thermo Fisher, Waltham, MA). Real-time PCR was performed based on SYBR-Green fluorescence (Bio-Rad, #170-8882AP Hercules, CA) and using specific primer sets for Arginase-1, iNOS, IL-6 and IL-10. Relative gene expression levels were normalized to GAPDH expression. The primer sets were following: 5’-CACGGCAGTGGCTTTAACCT and 5’-TGGCGCATTCACAGTCACTT for Arginase-1; 5’-TGGCCACCTTGTTCAGCTACG and 5’-GCCAAGGCCAAACACAGCATAC for iNOS; 5’-TCCAGTTGCCTTCTTGGGAC and 5’-GTACTCCAGAAGACCAGAGG for IL-6; 5’-GGTTGCCAAGCCTTATCGGA and 5’-ACCTGCTCCACTGCCTTGCT for IL-10; 5’-CAAGGTCATCCATGACAACTTTG and 5’-GTCCACCACCCTGTTGCTGTAG for GAPDH.

### Immunofluorescence staining and confocal microscopy

Tissue samples were embedded in optimal cutting temperature compound (OCT) and snap frozen in liquid nitrogen. Tissue sections (4 μm) were fixed in ethanol/acetic acid fixative solution for 2–10 min, and then were stained with anti-F4/80-PE (eBioscience), CD206-PerCP-Cy5.5 (Biolegend) overnight at 4°C in a humidified chamber. After three times of washes, slides were stained with DAPI and mounted with Prolong Gold mounting reagent (Invitrogen). Confocal imaging was performed using Leica SP5 II confocal microscope.

### Statistical analyses

Statistical analyses were performed using Student’s *t*-test for independent samples, with significance determined at P<0.05. All data and standard deviation (SD) were calculated and graphed by Microsoft Excel software (Microsoft, Redmond, WA).

## Results

### Biofilm formation in an *S*. *aureus*-infected PJI model

**Fig 1:**
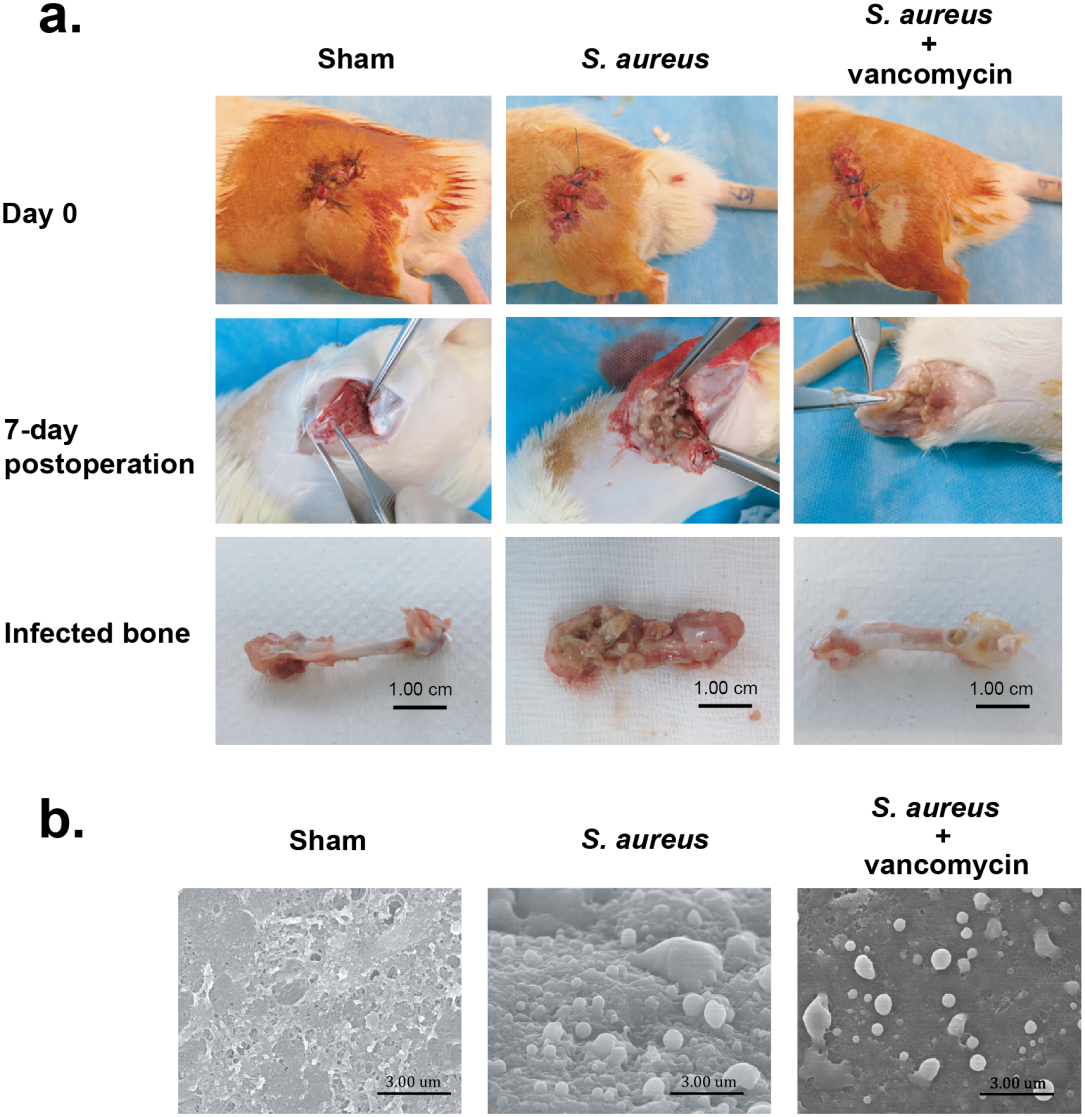
Surgical operation and *S*. *aureus* biofim formation in a rat PJI model. (a) Rats were divided into three groups including the sham group, the *S*. *aureus*-infected group and the *S*. *aureus*-infected group receiving vancomycin treatment. All rats were sacrificed 7 days after surgery and the treated femurs were taken. Notably, severe bone osteolytic lesion with pus formation was observed in the *S aureus*-infected femur, but less osteolytic lesion was detected in the vancomycin-treated infection femur. (b) Scanning electron microscopy images of biofilm formation in femurs at day 7 post-infection. Biofilm formation and *S*. *aureus* cocci were more evident in the femur lumen of the *S*. *aureus*-infected group than those of the vanomycin-treated infection group (original magnification ×10,000).

To mimic acute and chronic *S*. *aureus* biofilm infection *in vivo*, we initially established a PJI model in rats. In the PJI model, *S*. *aureus* (2×10^3^ CFU) were inoculated at a metallic orthopedic implant (Kirschner wire, 0.6 mm diameter x 0.5 cm) in rat femurs. In addition to the sham-operated and *S*. *aureus*-infected groups, the *S*. *aureus*-infected group receiving vancomycin (2 mg/kg) was also included in the experiment. At 7 days after surgery, all rats were sacrificed and the treated femurs were taken (Fig 1a). Compared to the sham-operated femurs, severe osteolytic bony destruction and formation of abscesses were observed in the *S*. *aureus*-infected femurs; however, vancomycin treatment substantially reduced the bony destruction caused by *S*. *aureus* infection (Fig 1a). By using scanning electron microscopy, biofilm formation and *S*. *aureus* cocci in the lumen of the femur were evidently detected in both *S*. *aureus*-infected groups, despite a lower extent of bacterial dissemination for the vancomycin-treated group (Fig 1b). These results indicated that our established PJI model in rats shows persistent biofilm infection and may allow further investigation of host immune responses to *S*. *aureus* biofilm infection *in vivo*.

### Significant expansion of MDSCs, total macrophages and M2-macrophages in the peripheral blood of rats during *S*. *aureus* biofilm infection

**Fig 2:**
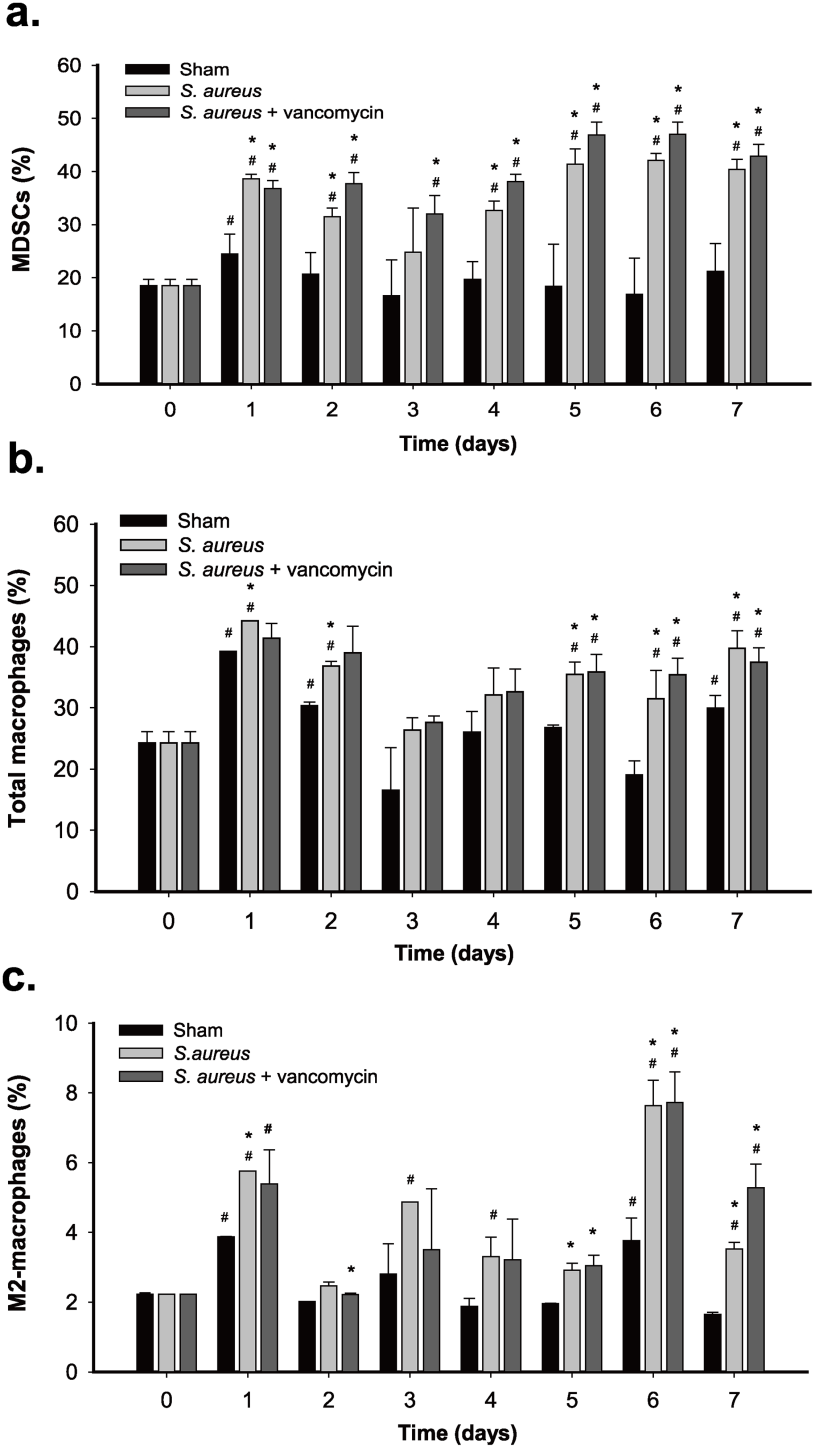
Expansion of MDSCs, total macrophages, and M2-macrophages during *S*. *aureus* infection in rats. The sham group, the *S*. *aureus*-infected group, and the *S*. *aureus*-infected group receiving vancomycin were included in the experiments and each group contained 4 rats. Blood samples were collected at different time points after operation. After lysis of the red blood cells, the remaining leukocytes were analyzed for the proportions of CD11bc^+^His48^+^ MDSCs (a), CD68^+^ macrophages (b), and CD68^+^CD206^+^ M2-macrophages (c) by flow cytometry. Symbol # indicates significant difference vs. day 0 (p < 0.01) and symbol * indicates significant difference vs. the shame group (p < 0.01).

To investigate the possible immunosuppressive effect caused by *S*. *aureus* biofilm infection *in vivo*, we examined if the proportions of the circulating MDSCs, total macrophages and M2-macrophages were changed during *S*. *aureus* infection in our PJI model. Blood samples from the sham-operated group, the untreated infection group, and the vancomycin-treated infection group were then collected at different time points after operation. Compared to blood samples obtained immediately prior to surgery (designated as day 0), blood samples collected from day 1 after surgery (or surgery plus *S*. *aureus* infection) exhibited higher proportions of MDSCs (CD11bc^+^HIS48^+^), macrophages (CD68^+^) and M2-macrophages (CD68^+^CD206^+^) in all three operated groups (Fig 2a–2c and S1 Fig), suggesting that surgical injury might contribute to systemic expansion of these immune cells. In the sham-operated group, the increased proportions of these immune cells detected on day 1 after surgery could decline gradually to normal levels on days 2 or 3 after surgery (Fig 2). However, when these immune cells were examined in the untreated infection group or in the vancomycin-treated infection group, we found that the proportions of MDSCs, total macrophages and M2-macrophages in the two *S*. *aureus*-infected groups were regularly higher than those in the sham-operated group at all postoperative times (Fig 2a–2c). These results suggested that *S*. *aureus* biofilm infection substantially stimulated the expansion of MDSCs, total macrophages, and M2-macrophages in the PJI model. Noteworthily, there were no significant differences in the expansion rates of MDSCs, total macrophages, and M2-macrophages between the two infected groups that received no treatment or vancomycin treatment, implicating that vancomycin treatment could not completely eliminate *S*. *aureus* biofilm infections during the experimental period.

### Treatment of mouse bone marrow cells with *S*. *aureus* biofilm increases the proportions of MDSCs, total macrophages and M2-macrophages *in vitro*

**Fig 3:**
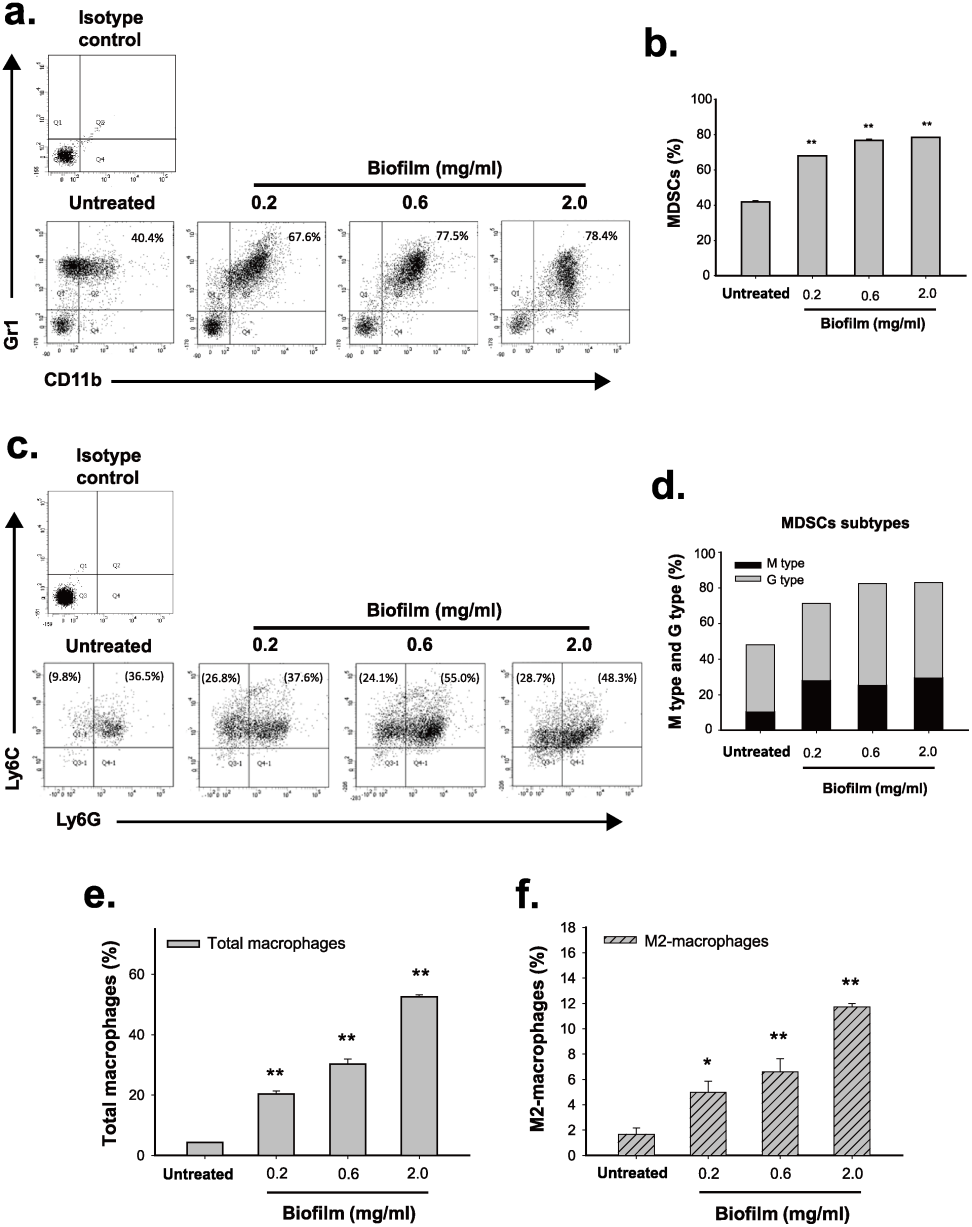
*S*. *aureus* biofilm triggers expansion of different immune cell types from mouse bone marrow cells *in vitro*. After coculture of mouse bone marrow cells (BMCs) with increasing concentrations (0.2, 0.6 and 2.0 mg/ml) of *S*. *aureus* biofilm for 48 hr, the proportions of CD11b^+^Gr1^+^ MDSCs (a and b), MDSC subsets including M-MDSCs (CD11b^+^Ly6C^high^Ly6G^-^) and G-MDSCs (CD11b^+^Ly6C^low^Ly6G^+^) (c and d), F4/80^+^ macrophages (e), as well as F4/80^+^CD206^+^ M2-macrophages (f) were evaluated by flow cytometry (N = 3). As noted, to examine the proportions of M-MDSCs and G-MDSCs, the CD11b^+^ cell populations were first gated from BMCs and then the proportions of Ly6C and Ly6G cells in the CD11b^+^ cell population were evaluated (c and d). Values in parentheses (c) represent the normalized percentages of individual MDSC subsets in BMCs. *p < 0.01, **p < 0.001.

Since the circulating MDSCs, total macrophages, and M2-macrophages could be derived from bone marrow precursor cells (BMCs), we attempted to investigate whether *S*. *aureus* biofilm was able to stimulate the expansion of these immune cell types from BMCs *in vitro*. Here, we developed an *in vitro* coculture system in which the freshly isolated mouse BMCs were plated on the bottom of transwell cell culture system and then co-cultured with *S*. *aureus* biofilms loaded in the transwell cell culture insert (0.4 μm pore size). Notably, mouse BMCs (C57BL/6J) rather than rat BMCs were used in the testing because cell-surface markers for diverse immune cell types are better characterized in mouse. After treatment of mouse BMCs with increasing amounts (0.2, 0.6 and 2.0 mg/ml) of *S*. *aureus* biofilm for 48 hr, we found that the proportions of MDSCs (CD11b^+^Gr-1^+^) were significantly increased from 40% to about 67–80% along with the doses of *S*. *aureus* biofilm (Fig 3a and 3b). The MDSC subtypes, including M-MDSCs (CD11b^+^Ly6C^high^Ly6G^-^) and G-MDSCs (CD11b^+^Ly6C^low^Ly6G^+^), were then evaluated in the experiments. Interestingly, *S*. *aureus* biofilm at the low concentration (0.2 mg/ml) sufficiently elicited a marked increase in the proportion of M-MDSCs (from 9.8% to 26.8%), but not the proportion of G-MDSCs (from 3.6.5% to 37.6%), from the treated BMCs (Fig 3c and 3d). As compared to the untreated group, a mild increase in the proportion of G-MDSCs was detected only when BMCs were treated with *S*. *aureus* biofilm at high concentrations (0.6 and 2.0 mg/ml), resulting in an increase from 36.5% to 55% or 48.3% (Fig 3c and 3d). These results implicated that *S*. *aureus* biofilm differentially promotes the expansion of M-MDSCs and G-MDSCs.

In addition to MDSCs, the proportions of total macrophages and M2-macrophages were also measured in the experiments. We consistently found that *S*. *aureus* biofilm markedly increased the proportions of total macrophages (F4/80^+^) and M2-macrophages (F4/80^+^CD206^+^) in a dose-dependent manner (Fig 3e and 3f and S2 Fig). In agreement with the PJI model in rats, our *in vitro* coculture system reflected that *S*. *aureus* biofilm could elicit the expansion of MDSCs, total macrophages, and M2-macrophages from BMCs.

### *S*. *aureus* biofilm promotes the differentiation of MDSCs into M2-macrophages *in vitro*

**Fig 4:**
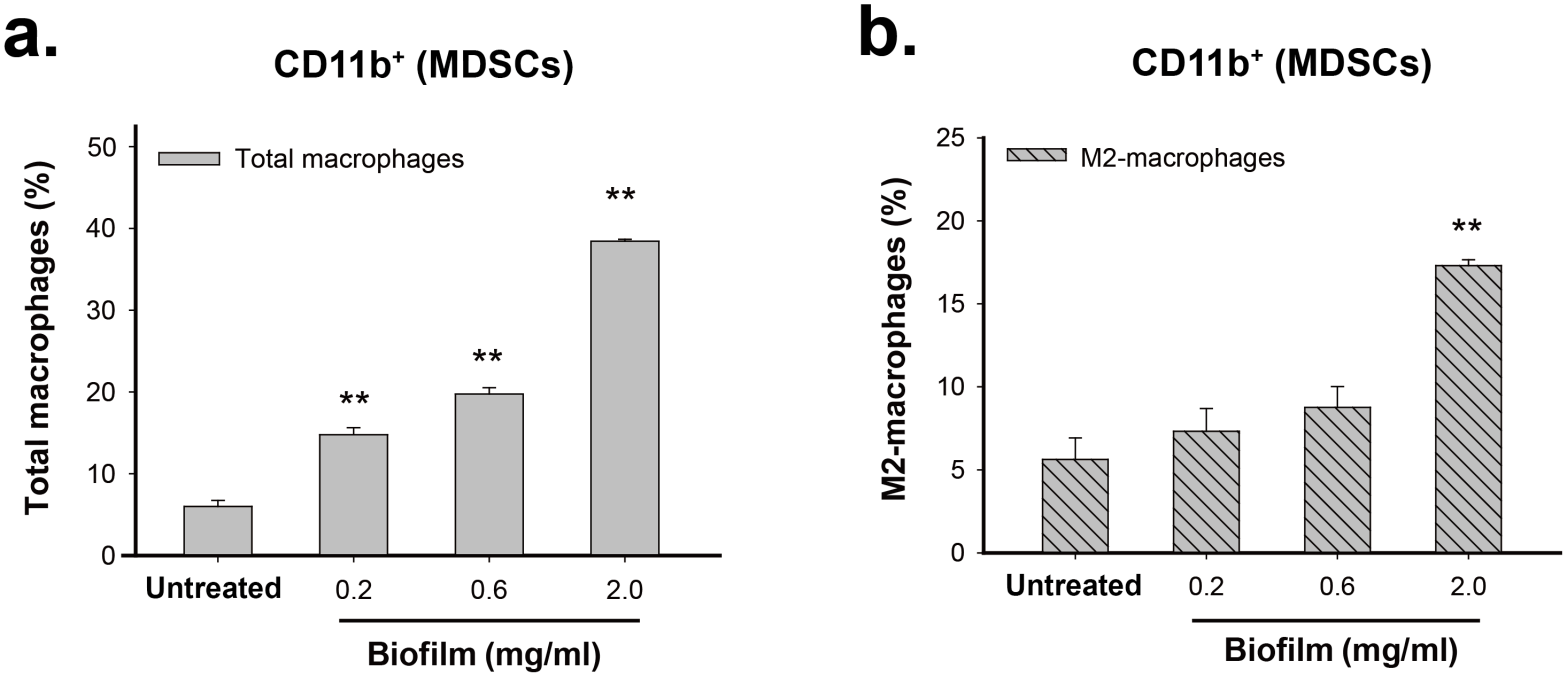
*S*. *aureus* biofilm is capable of stimulating the conversion of the CD11b-positive MDSCs into macrophages or M2-macrophgates *in vitro*. The isolated CD11b-positive MDSCs were cocultured with elevated amounts (0.2, 0.6 and 2.0 mg/ml) of *S*. *aureus* biofilm for 48 hr. The proportions of total macrophages (F4/80^+^) (a) and M2-macrophages (F4/80^+^CD206^+^) (b) in the CD11b-positive cell population were measured by flow cytometry. **p < 0.001.

**Fig 5:**
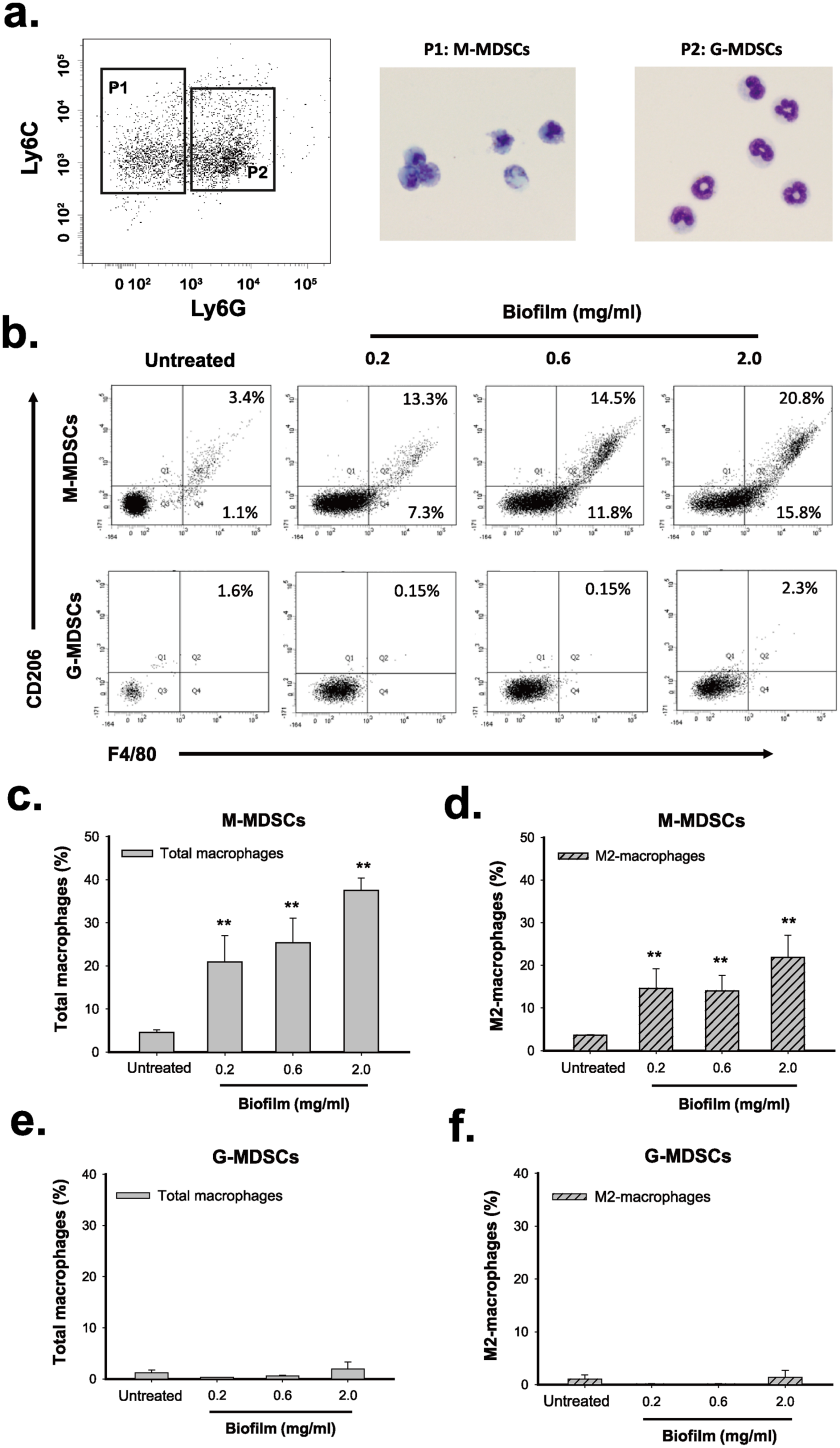
*S*. *aureus* biofilm promotes differentiation of M-MDSCs but not G-MDSCs into macrophages or M2-macrophages *in vitro*. (a) Morphological characterization of the sorted M-MDSCs (CD11b^+^Ly-6C^+^Ly-6G^-^) and G-MDSCs (CD11b^+^Ly-6C^low^Ly-6G^+^). Giemsa-stained M-MDSCs (P1) and G-MDSCs (P2) were examined by light microscopy. (b) M-MDSCs and G-MDSCs were individually co-cultured with *S*. *aureus* biofilms for 72 hr. The biofilm-treated cells were subsequently analyzed for the expression of F4/80 and CD206 by flow cytometry. (c) Changes in the proportion of F4/80-positive macrophages in the sorted M-MDSCs after treatment with *S*. *aureus* biofilm (N = 3). (d) Changes in the proportion of M2-macrophages (F4/80^+^CD206^+^) in the sorted M-MDSCs after biofilm treatment (N = 3). (e and f) Treatment of G-MDSCs with *S*. *aureus* biofilm failed to affect the proportions of F4/80^+^ cells (macrophages) and F4/80^+^CD206^+^ cells (M2-macrophages) (N = 3). **p < 0.001.

Since MDSCs are an immature population of myeloid cells, we then investigated whether MDSCs could be programmed toward M2-macrophages in response to *S*. *aureus* biofilms. To do the experiments, we first isolated the CD11b-positive cells from mouse BMCs using CD11b MicroBeads, which correspond to MDSC population (S3 Fig and see Discussion). We found that treatment of the isolated CD11b-positive MDSCs with increasing amounts of *S*. *aureus* biofilm caused significant increases in the proportions of total macrophages (F4/80^+^) and M2-macrophages (F4/80^+^CD206^+^) in a dose-dependent manner (Fig 4a and 4b). These results suggested that the CD11b-positive MDSCs had the potential to convert into M2-macrophages upon exposure to *S*. *aureus* biofilm. To further elucidate the conversion of these immune cells, two MDSC subsets (M-MDSCs and G-MDSCs) were sorted from the CD11b-positive MDSCs by cell sorter (Fig 5a). These two isolated MDSC subpopulations, M-MDSCs (CD11b^+^Ly6C^high^Ly6G^-^) and G-MDSCs (CD11b^+^Ly6C^low^Ly6G^+^), were then cocultured with *S*. *aureus* biofilm. Specially, like the CD11b-positive MDSCs, treatment of the sorted M-MDSC cell population (Fig 5a, P1) with *S*. *aureus* biofilm also exhibited increased proportions of total macrophages (F4/80^+^) and M2-macrophages (F4/80^+^CD206^+^) along with an increasing amount of biofilm (Fig 5b top, Fig 5c and 5d). In contrast, the coculture of the sorted G-MDSCs (Fig 5a, P2) with *S*. *aureus* biofilm did not cause expansion of macrophages (F4/80^+^) and M2-macrophages (F4/80^+^CD206^+^) (Fig 5b bottom, Fig 5e and 5f). These results indicated that *S*. *aureus* biofilm is capable of promoting the conversion of M-MDSCs (CD11b^+^Ly6C^high^Ly6G^-^) into M2-macrophages (F4/80^+^CD206^+^) *in vitro*.

### *S*. *aureus* biofilm enhances the immunosuppressive activity of BMCs and the CD11b-positive MDSCs *in vitro*

**Fig 6:**
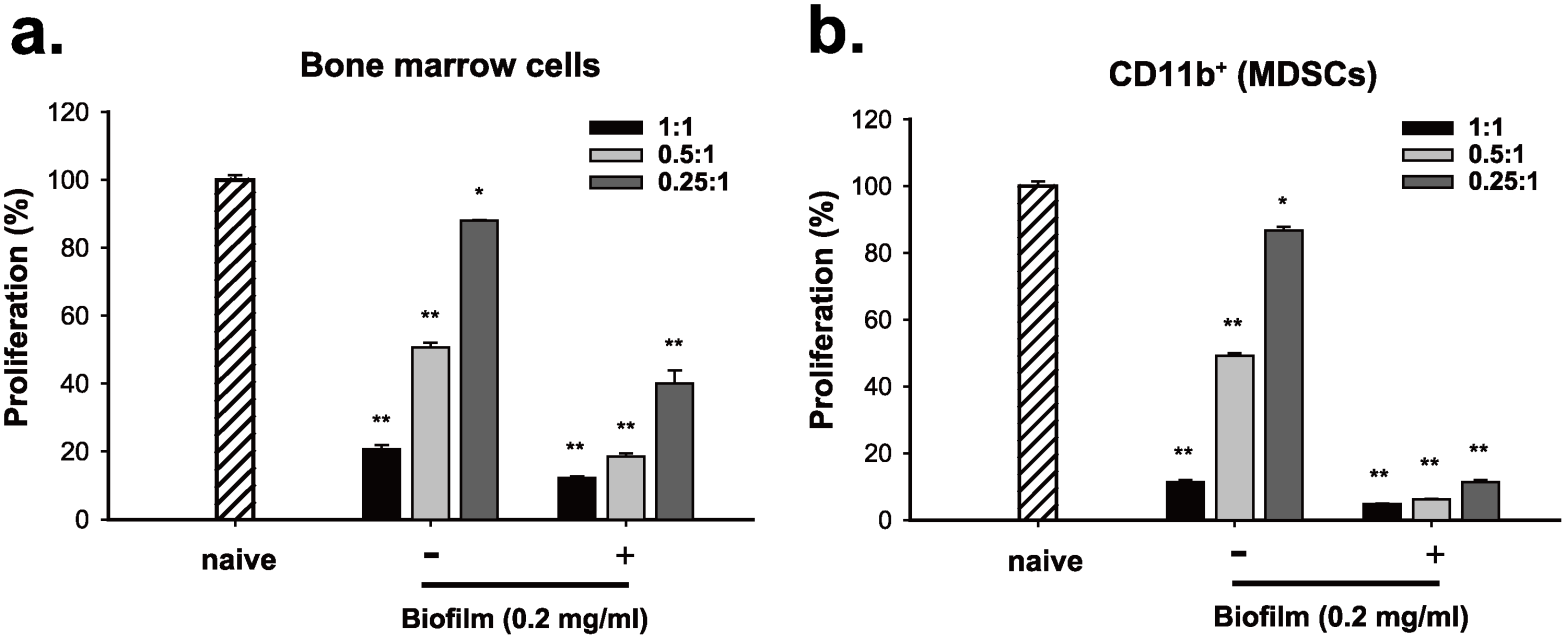
*S*. *aureus* biofilm augments the immunosuppressive activity of the cultured BMCs and the CD11b-positive population *in vitro*. (a) Mouse BMCs left untreated or treated with *S*. *aureus* biofilm (0.2 mg/ml) for 48 hr were cocultured with activated CFSE-labeled T cells at different ratios (1:1, 0.5:1 and 0.25:1). The proliferation of CFSE-labeled T cells was examined by flow cytometry (N = 3). Proliferation of T cell control stimulated by anti-CD3/CD28 beads only was set as 100% (naive) in the experiment. (b) The CD11b-positive cell populations isolated from the untreated or biofilm-treated BMCs were used to coculture with activated CFSE-labeled T cells at different ratios (1:1, 0.5:1 and 0.25:1). The proliferation of CFSE-labeled T cells was examined by flow cytometry (N = 3). *p < 0.01, **p < 0.001.

In addition to the expansion of the immunosuppressive cells induced by *S*. *aureus* biofilm, we next determined whether *S*. *aureus* biofilm substantially affected their immunosuppressive function *in vitro*. Herein, mouse BMCs were first left untreated or treated with *S*. *aureus* biofilm (0.2 mg/ml) for 48 hr. When the untreated or biofilm-treated BMCs were cocultured with activated CFSE-labeled T cells at different ratios (1:1, 0.5:1 and 0.25:1), we found that biofilm-treated BMCs evidently displayed greater suppressive activities on T cell proliferation as compared to the untreated BMCs (Fig 6a). Since biofilm treatment could promote MDSC expansion, equal numbers of the CD11b-positive MDSCs isolated from the untreated or biofilm-treated BMCs were used to compare their immunosuppressive function. In T cell suppression assays, we consistently found that the CD11b-positive MDSCs isolated from biofilm-treated BMCs had a much stronger immunosuppressive activity than those from untreated BMCs (Fig 6b).

**Fig 7:**
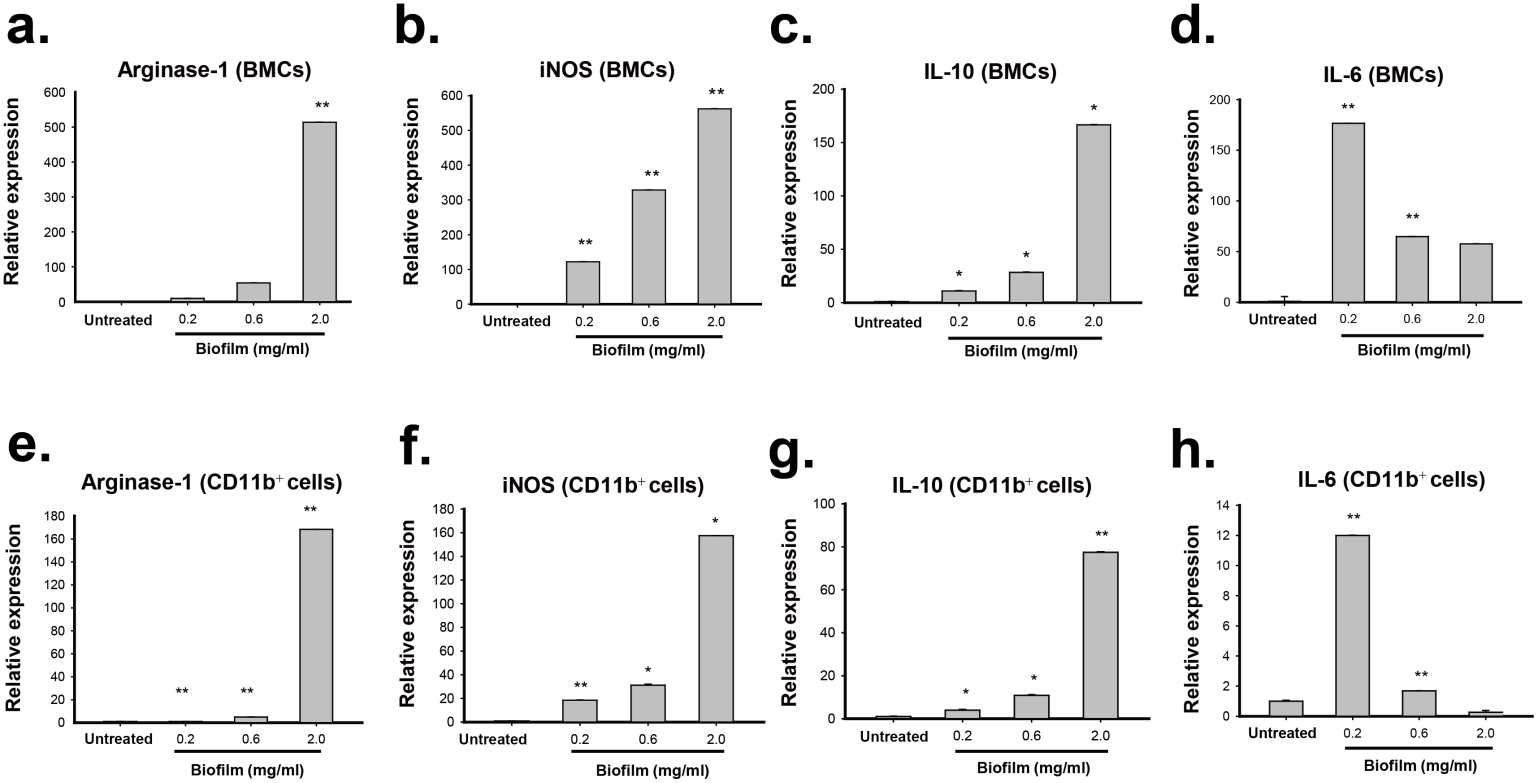
Expression of Arginase-1, iNOS, IL-10 and IL-6 in BMCs or in the CD11b-positive MDSCs after exposure to *S*. *aureus* biofilm. (a-d) Mouse BMCs were treated with different amounts (0.2, 0.6 and 2.0 mg/ml) of *S*. *aureus* biofilm for 48 hr. RNA samples extracted from the untreated or biofilm-treated BMCs were used for analyzing the expression levels of Arginase-1, iNOS, IL-10 and IL-6 by quantitative RT-PCR. (e-h) The expression levels of Arginase-1, iNOS, IL-10 and IL-6 mRNAs were quantified among the CD11b-positive cell populations isolated from the biofilm-treated BMCs as described above. *p < 0.01, **p < 0.001.

To further confirm the elevated immunosuppressive activity in these tested effector cells caused by biofilm, the expression patterns of various anti-inflammatory-related genes including Arginase-1, iNOS, IL-6 and IL-10 were measured in the untreated or biofilm-treated cells (Fig 7). Quantitative RT-PCR analysis showed that BMCs treated with increasing amounts of *S*. *aureus* biofilm substantially displayed enhanced expressions of Arginase-1, iNOS and IL-10 mRNAs (Fig 7a–7c), supporting that biofilm-treated BMCs have higher immunosuppressive activities than the untreated BMCs. Moreover, when gene expression patterns were compared between the CD11b-positive MDSCs isolated from the untreated and biofilm-treated BMCs, the levels of Arginase-1, iNOS and IL-10 mRNAs were also particularly higher in biofim-treated MDSC populations (Fig 7e–7g). Notably, we noticed that the levels of IL-6, which has both pro- and anti-inflammatory properties [29], could be abundantly induced in BMCs or in the CD11b-positive MDSCs when exposed to a low concentration of biofilm (0.2 mg/ml). However, treatment of these cell populations with biofilm at high concentrations (0.6 and 2.0 mg/ml) caused only a moderate increase in the levels of IL-6 as compared to the untreated controls (Fig 7d and 7h).

**Fig 8:**
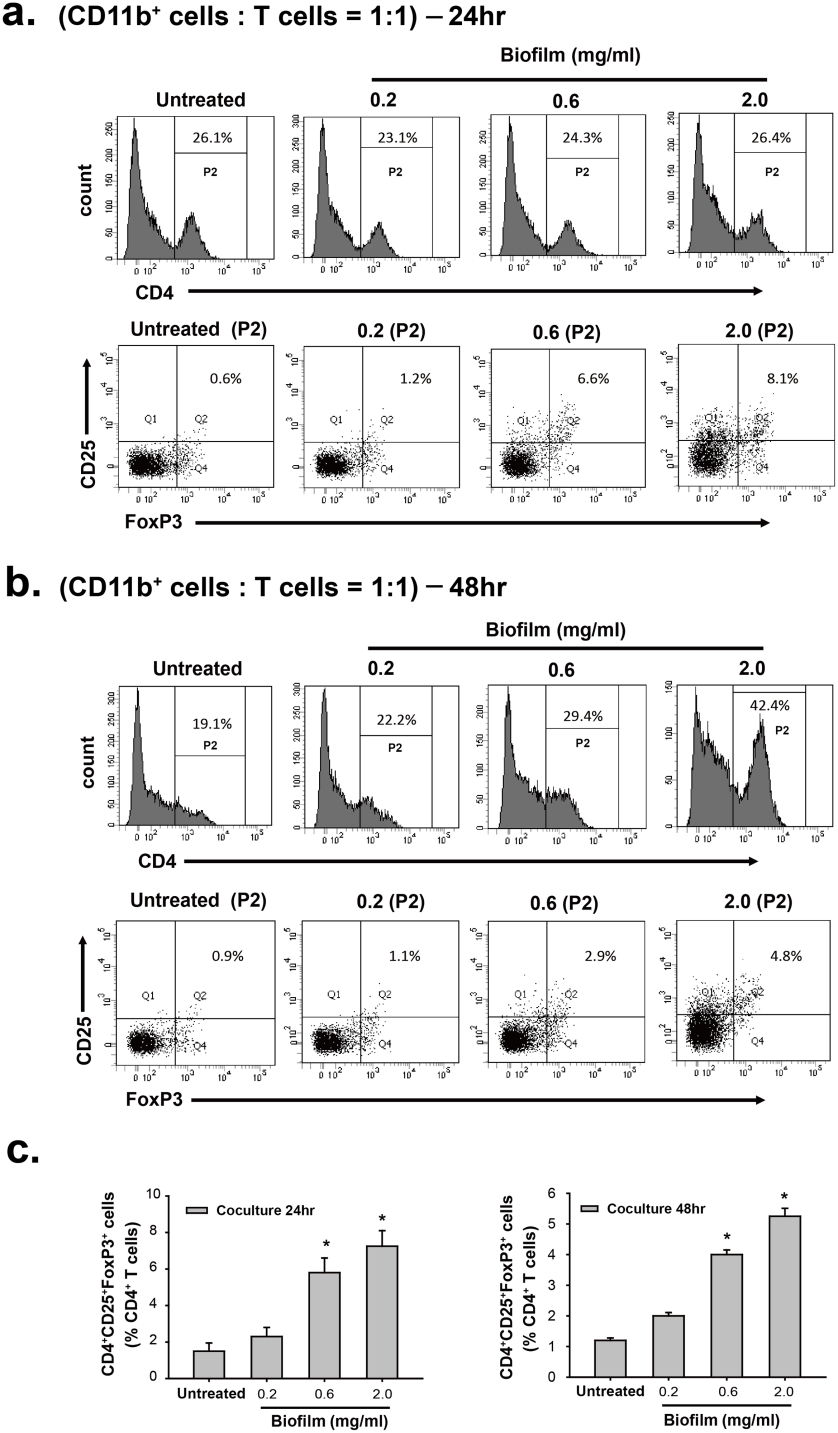
*S*. *aureus* biofilm is able to promote expansion of CD4^+^CD25^+^Foxp3^+^ Tregs from CD4^+^ lymphocytes through modulation of MDSC function *in vitro*. (a and b) The CD11b-positive MDSC fractions were individually isolated from BMCs that were untreated or treated with different amounts (0.2, 0.6 and 2.0 mg/ml) of biofilm. The isolated CD11b-positive MDSCs were subsequently cocultured with spleen T cells (activated by anti-CD3/CD28 beads) at a 1:1 ratio. After 24- or 48-hr coculture, the frequency of CD4^+^CD25^+^Foxp3^+^ T cells was analyzed by flow cytometry using a Treg Flow kit. (c and d) Bars indicate the percentages of CD4^+^CD25^+^Foxp3^+^ T cells out of CD4^+^ lymphocytes in coculture experiments as described above (N = 2).

In addition to MDSCs and M2-macrophages, other immunosuppressive cells such as CD4^+^CD25^+^Foxp3^+^ regulatory T cells (Tregs) could possibly involve the immune modulation during *S*. *aureus* infection [30–32]. Since *S*. *aureus* biofilm significantly enhanced the immunosuppressive activity of MDSCs to inhibit T cell responses, we here attempted to determine whether the biofilm-treated MDSCs could modulate expansion of Tregs from spleen T cells *in vitro*. To do the experiments, the CD11b-positive cell populations (MDSCs) were isolated from mouse BMCs that were untreated or treated with increasing amounts of biofilm for 48 hr. The isolated MDSC populations were subsequently co-cultured at the 1:1 ratio with spleen T cells that were activated by anti-CD3/CD28 beads. As compared to the coculture of spleen T cells with the untreated MDSCs, we unexpectedly found that the percentages of CD4^+^CD25^+^Foxp3^+^ Tregs in CD4^+^ lymphocytes were significantly elevated in the cocultures of spleen T cells with the biofilm-treated MDSCs after 24- or 48-hr coculture (Fig 8). Furthermore, the isolated MDSCs undergoing treatment for biofilm at higher concentrations had higher capacity to increase the percentages of CD4^+^CD25^+^Foxp3^+^ Tregs in CD4^+^ lymphocytes (Fig 8). These results strongly suggested that biofilm-treated MDSCs could suppress immunity through multiple different mechanisms.

### Recruitment of the transferred EGFP-labeled, CD11b-positive MDSCs into the site of *S*. *aureus* infection

**Fig 9:**
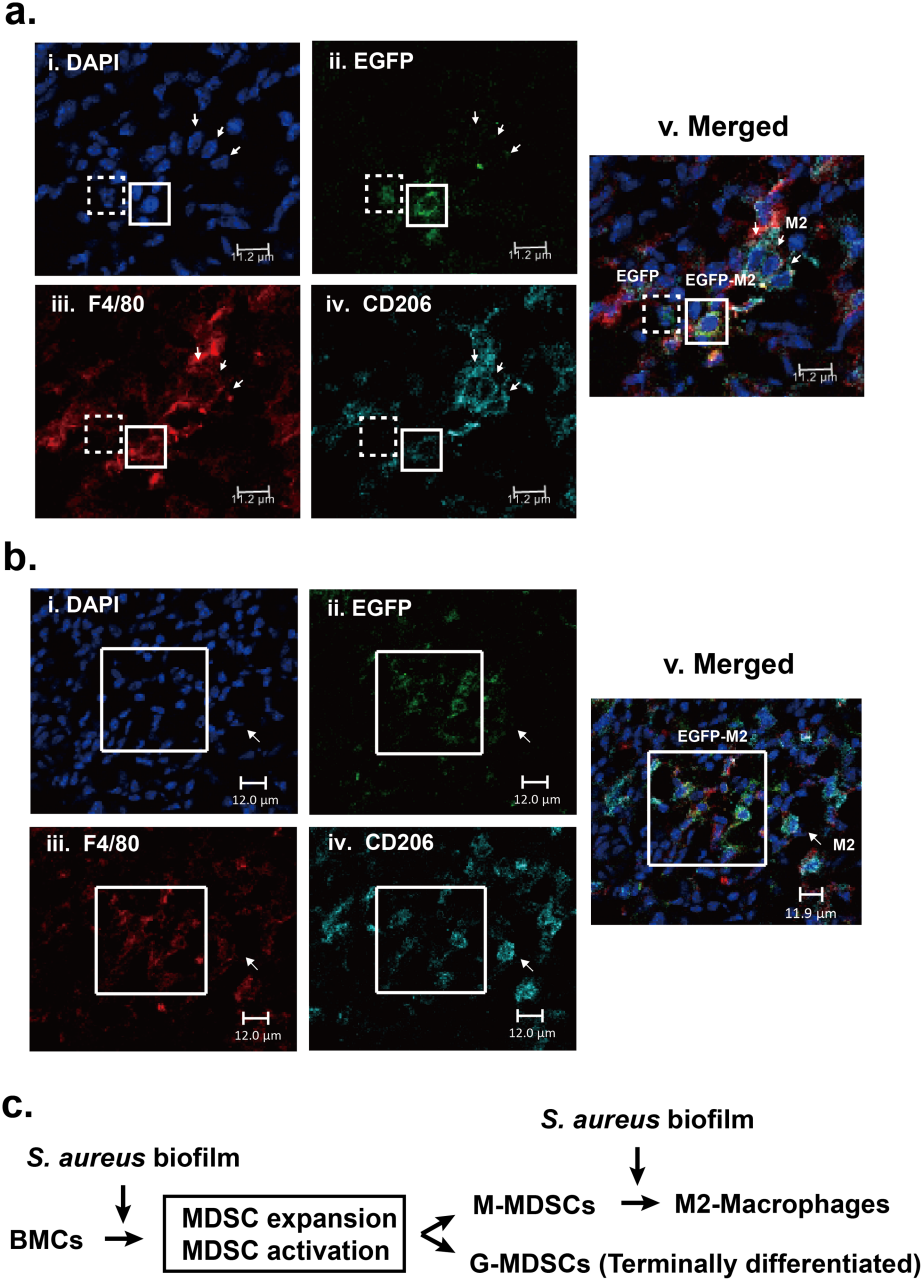
Recruitment and polarization of exogenous EGFP-expressing, CD11b-positive cells during *S*. *aureus* biofilm infection in mice. (a and b) Representative confocal microscopic images showing the recruitment and polarization of the EGFP-expressing cells in tissues near the *S*. *aureus* infection sites. Tissues near the *S*. *aureus* infected site were taken, frozen in -80°C, cryosectioned and stained with F4/80-PE (a and b, iii) and CD206-PerCP antibody (a and b, iv). White boxes indicate the EGFP^+^F4/80^+^CD206^+^ cells, and dashed boxes indicate the EGFP^+^F4/80^-^CD206^-^ cells (probably MDSCs). Notably, only a very small proportion (<15%) of EGFP-positive cells were detected as the EGFP^+^F4/80^-^CD206^-^ or EGFP^+^F4/80^+^CD206^-^ phenotype in the infected sites. Moreover, cells double positive for F4/80 and CD206 (arrows; endogenous M2-macrophages) were commonly detected in the infected sites. (c) A model for the development and polarization of MDSCs during *S*. *aureus* biofilm stimulation.

To study whether the circulating MDSCs could be recruited into the site of *S*. *aureus* infection in femurs and converted into M2-macrophages *in vivo*, we here employed a mouse model of PJIs to address these questions. In this model, C57BL/6J mice were infected with *S*. *aureus* for 7 days, and then 1×10^7^ exogenously EGFP-labeled, CD11b-positive MDSCs were intravenously injected into the infected mice. Twenty-four hours later, tissues near the *S*. *aureus*-infected site were taken, embedded, frozen in -80°C, cryosectioned, and then stained with anti-F4/80 and anti-CD206 antibodies. Confocal microscopy analysis revealed that EGFP-positive cells were indeed recruited into the site of *S*. *aureus* infection (Fig 9a and 9b, i and ii). Especially, about 85% of EGFP-positive cells were positive for both F4/80 and CD206 (Fig 9b, iii and iv). These results strongly suggested that *S*. *aureus* biofilm infection could promote the conversion of MDSCs to M2-macrophages (EGFP^+^F4/80^+^CD206^+^) *in vivo* (Fig 9). Additionally, we here noticed that the overall number of M2-macrophages (F4/80^+^CD206^+^) was much higher than that of F4/80^+^CD206^-^ cells (probably pro-inflammatory macrophages) present in *S*. *aureus*-infected sites (Fig 9a and 9b, iii and iv). A possible pathway for the development and polarization of MDSCs during *S*. *aureus* biofilm stimulation is proposed in Fig 9c.

## Discussion

*S*. *aureus* biofilm infection in PJIs is the major complication in joint reconstruction. The formation of bacterial biofilm is important not only for protection against antibiotic treatment but also for evasion of host immune responses. In the latter case, *S*. *aureus* biofilm that interferes with the immune responses involves accumulation of both MDSCs and M2-macrophages in infected sites [12, 33–35]. In this report, we confirmed that *S*. *aureus* biofilm triggers the expansion and activation of MDSCs and M2-macrophages in our established *in vitro* and *in vivo* models. For MDSC expansion, we found that *S*. *aureus* biofilm preferentially induces the expansion of M-MDSCs but not G-MDSCs. Importantly, our results support that M-MDSCs have the potential to further differentiate into M2-macrophages in the presence of *S*. *aureus* biofilm. To our knowledge, this is the first study showing the relationship between MDSCs and M2-macrophages in *S*. *aureus* biofilm infection. Overall, our findings provide a better understanding of the development of these immunosuppressive cells during *S*. *aureus* biofilm infection.

Using our established PJI model in rats, two *S*. *aureus*-infected groups that received no treatment or vancomycin treatment were evaluated. Although vancomycin treatment greatly reduced bone infection caused by *S*. *aureus* (Fig 1a), *S*. *aureus* infection might persistently damage the infected femur even standard treatment with antibiotics. Particularly, we found that aggregates of *S*. *aureus* embedded with a self-produced extracellular matrix, which adhere to bone or prosthesis surface, were still observed in the vancomycin-treated group by scanning electron microscopy (Fig 1b). On the other hand, we did not detect significant differences in the expansion rates of the circulating MDSCs, macrophages, and M2-macrophages in the peripheral blood between the two *S*. *aureus*-infected groups that received no treatment or vancomycin treatment (Fig 2). Our results emphasize that *S*. *aureus* biofilm infection in PJIs is still a great challenge for orthopedic surgeons, and antibiotic treatment may be not so effective to eliminate the persistent bacterial infection.

In addition to a rat PJI model, we here successfully established an *in vitro* coculture system to study effects of *S*. *aureus* biofilm on the expansion of MDSCs, total macrophages and M2-macrophages from freshly isolated BMCs (Fig 3). Originally, we attempted to coculture BMCs with *S*. *aureus* biofilm directly. However, the direct contact between BMCs and *S*. *aureus* biofilm led to rapid cell death. To avoid the over-reaction of immune cells, *S*. *aureus* biofilms in our *in vitro* system were loaded in transwell cell culture inserts (0.4 μm pore size) and cocultured with BMCs. Consistent with the results found in our rat PJI model, we showed that treatment of BMCs with *S*. *aureus* biofilm in our *in vitro* system stimulated the expansions of MDSCs (CD11b^+^Gr1^+^), total macrophages (F4/80^+^) and M2-macrophages (F4/80^+^CD206^+^) (Fig 3). Most importantly, a dose-response relationship between *S*. *aureus* biofilm and the expansions of these immune cells could be established in the *in vitro* system.

MDSCs are composed of two subsets, M-MDSCs and G-MDSCs. Evidence has shown that these two subsets may manifest different functions under different circumstances [11]. Although earlier studies by Hem *et al*. [14] have reported that MDSCs function as a central contributor to mediate immunosuppression during *S*. *aureus* biofilm infection, the expansion and polarization of MDSC subsets in response to *S*. *aureus* biofilm remained poorly understood. Using our *in vitro* coculture system, we clearly showed that although both M-MDSCs and G-MDSCs could expand upon exposure to *S*. *aureus* biofilm, the expansion of M-MDSCs was much greater than G-MDSCs (Fig 3c and 3d). The maximal expansion of M-MDSC population in BMCs could be easily achieved after exposure to a low concentration (0.2 mg/ml) of *S*. *aureus* biofilm (Fig 3c and 3d; from 9.8% to 26.8%); however, higher concentrations (0.6 and 2.0 mg/ml) of *S*. *aureus* biofilm did not further elicit M-MDSC expansion. There are two possibilities that may explain these results. One possibility is that a low concentration of *S*. *aureus* biofilm may be already sufficient to confer the full induction of M-MDSC expansion from BMCs. The other possibility is that, in response to high concentrations of biofilm, the increased proportions of M-MDSCs may be able to further differentiate into another cell types, consequently maintaining a fixed level of M-MDSCs. In the study, we did find that M-MDSCs have the capacity to differentiate into M2-macrophages during *S*. *aureus* biofilm stimulation (Figs 5 and 9).

Although MDSCs in mice are commonly characterized as CD11b^+^Gr1^+^ cells, we noticed that almost all of CD11b-positive cells (up to 97%) freshly isolated from mouse bone marrow cells co-expressed Gr1 (or Ly6C/Ly6G) in our experiments (S3 Fig). This information would be helpful because the use of the CD11b-positive cells as a major MDSC cell population avoids tedious cell sorting processes that often result in cell membrane damage. Treatment of MDSCs (CD11b^+^) with *S*. *aureus* biofilm substantially increased the proportions of M2-macrophageswe *in vitro*. Furthermore, we revealed that M-MDSCs (CD11b^+^Ly6C^high^Ly6G^-^) but not G-MDSCs (Ly6C^low^Ly6G^+^) polarize toward M2-macrophges (F4/80^+^CD206^+^) in response to *S*. *aureus* biofilm (Fig 5b and 5d). Consistent with the *in vitro* studies, our *in vivo* mouse model also revealed that about 85% of exogenously injected EGFP-expressing CD11b-positive MDSCs were skewed toward M2-macrophages in surrounding soft tissue of infected bone (Fig 9).

To support the immunosuppressive function of these immune cells, both BMCs and MDSCs (CD11b^+^) untreated or treated with biofilm were co-cultured with activated T cells (Fig 6). Our results consistently showed that biofilm treatment significantly enhanced the suppressive activity of BMCs or the CD11b-positive MDSCs on T cell proliferation. Furthermore, quantitative RT-PCR analysis also revealed that the biofilm-treated cell populations abundantly expressed anti-inflammatory mediators, including Arginase-1, iNOS and IL-10 (Fig 7). In addition to these anti-inflammatory mediators, we noticed that IL-6 was markedly stimulated in BMCs or the MDSC (CD11b^+^) population after exposure to a low concentration of biofilm (0.2 mg/mL); however, at higher concentrations of biofilm the induction of IL-6 trended toward a modest increase (Fig 7d and 7h). It may be not so surprising because IL-6 is previously known to have both pro- and anti-inflammatory properties [29]. Since *S*. *aureus* biofilm enhanced the immunosuppressive activity of MDSCs to inhibit T cell responses, we additionally investigated whether the biofilm-treated MDSCs could modulate Treg cells, another cell type with immunosuppressive function. When compared to the coculture of spleen T cells with the untreated MDSCs, we unexpectedly found that the proportions of CD4^+^CD25^+^Foxp3^+^ Tregs in CD4^+^ lymphocytes could be significantly increased in the cocultures of spleen T cells with the biofilm-treated MDSCs (Fig 8). These results therefore suggested that biofilm-treated MDSCs could suppress immunity through multiple different ways. Taken together, our findings support that *S*. *aureus* biofilm mediate both MDSC expansion and activation.

In conclusion, since recalcitrant PJIs are still a difficult challenge for orthopaedic surgeons, a detailed understanding of the relationship between MDSCs, M2-macrophages and biofilms may further expand the possibility of immunotherapies in treating PJIs in the future.

## Supporting information

S1 Fig Representative flow cytometry plots showing expansions of MDSCs, total macrophages, and M2-macrophages during *S*. *aureus* infection in rats. Rat blood samples were collected from the sham group, the untreated infection group, and the vancomycin-treated infection group at different time points after operation. After lysing the red blood cells, the remaining leukocytes were analyzed by flow cytometry for the proportions of CD11bc^+^His48^+^ MDSCs (a), and the proportions of CD68^+^ macrophages and CD68^+^CD206^+^ M2-macrophages (b).
(TIF)

S2 Fig Flow cytometry analysis of activation of macrophages and M2 macrophages from BMCs after treatment with *S*. *aureus* biofilm *in vitro*. Mouse BMCs were cultured with increasing concentrations (0.2, 0.6 and 2.0 mg/ml) of *S*. *aureus* biofilm for 48 hr. Representative flow cytometry histograms show the expansion of F4/80^+^ macrophages and F4/80^+^CD206^+^ M2-macrophages from BMCs caused by *S*. *aureus* biofilm.
(TIF)

S3 Fig Almost all of the CD11b-positive cells isolated from BMCs reveal a coexpression of Gr1 (or Ly6C/Ly6G), which correspond to the double-positive MDSCs (CD11b^+^Gr1^+^). (a) The CD11b-positive cell population was sorted from bone marrow cells by BD FACSAria Fusion cell sorter, and then subjected to flow cytometric analysis. Up to 99% of the CD11b-positive cells co-expressed Gr1. (b) The CD11b-positive cell population was isolated from bone marrow cells using CD11b magnetic beads (STEMCELL^™^). Up to 97% of the CD11b-positive cells co-expressed either Ly6C or Ly6G (two components of Gr1).
(TIF)
