# Supplementary figures and images for: Staphylococcus aureus biofilm elicits the expansion, activation and polarization of myeloid-derived suppressor cells in vivo and in vitro
Source: PLoS One. 2017 Aug 16;12(8):e0183271. doi: 10.1371/journal.pone.0183271 (PMC5559065; doi:10.1371/journal.pone.0183271)

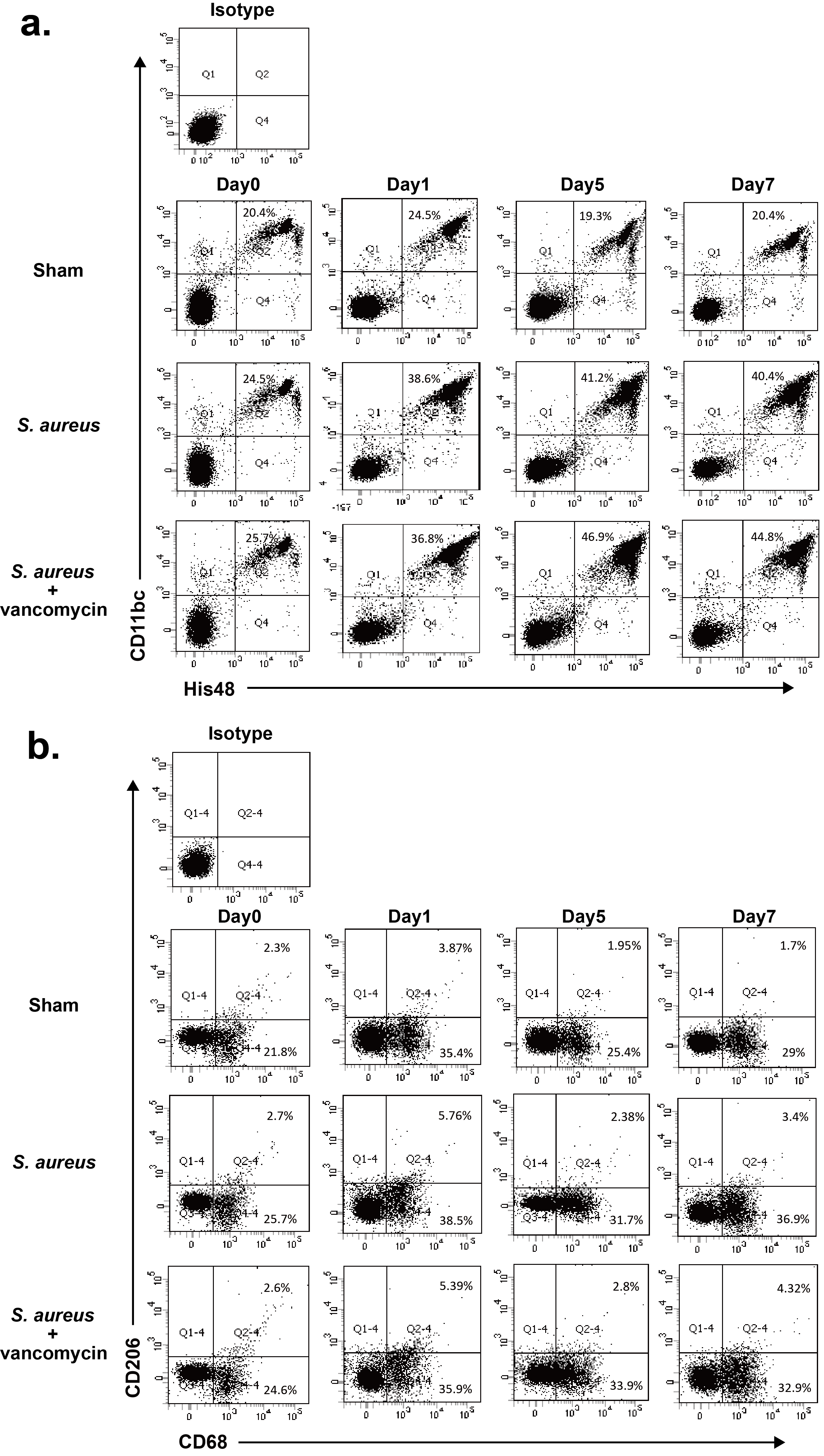

Supplement: S1 Fig — Rat blood samples were collected from the sham group, the untreated infection group, and the vancomycin-treated infection group at different time points after operation. After lysing the red blood cells, the remaining leukocytes were analyzed by flow cytometry for the proportions of CD11bc+His48+ MDSCs (a), and the proportions of CD68+ macrophages and CD68+CD206+ M2-macrophages (b). (TIF) [file pone.0183271.s001.tif]

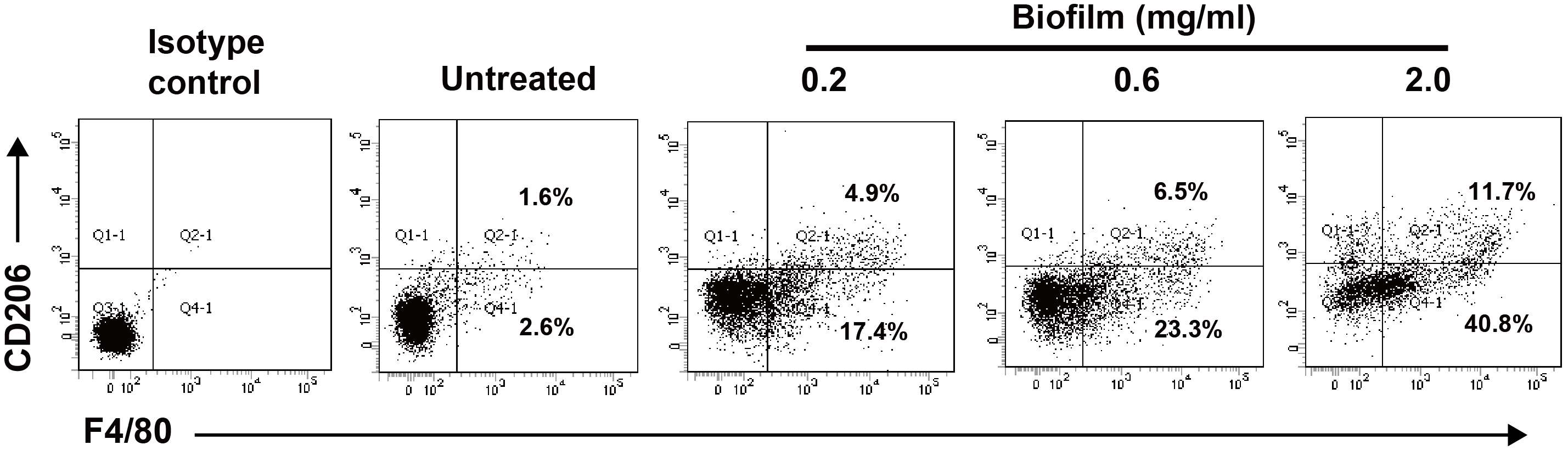

Supplement: S2 Fig — Mouse BMCs were cultured with increasing concentrations (0.2, 0.6 and 2.0 mg/ml) of S. aureus biofilm for 48 hr. Representative flow cytometry histograms show the expansion of F4/80+ macrophages and F4/80+CD206+ M2-macrophages from BMCs caused by S. aureus biofilm. (TIF) [file pone.0183271.s002.tif]

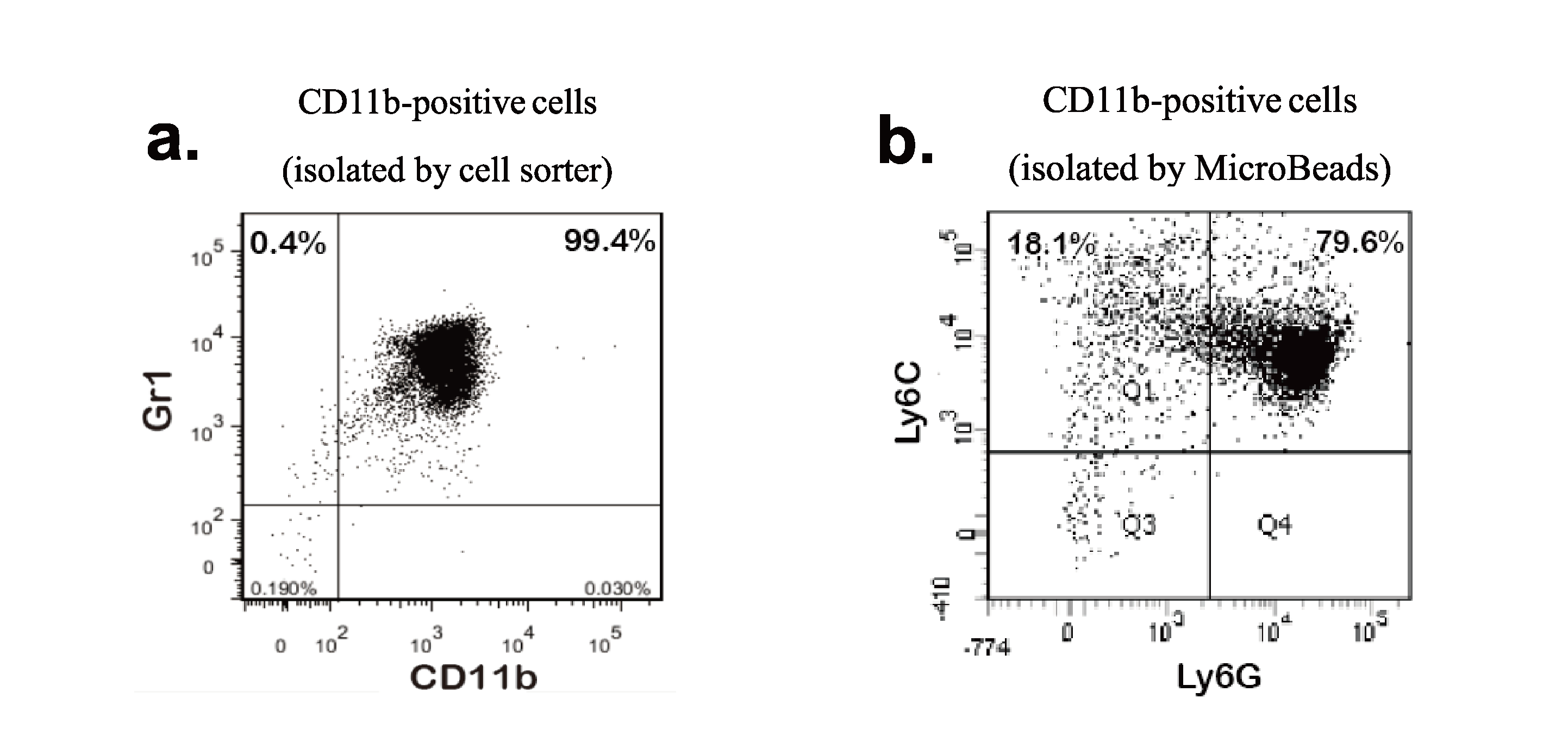

Supplement: S3 Fig — (a) The CD11b-positive cell population was sorted from bone marrow cells by BD FACSAria Fusion cell sorter, and then subjected to flow cytometric analysis. Up to 99% of the CD11b-positive cells co-expressed Gr1. (b) The CD11b-positive cell population was isolated from bone marrow cells using CD11b magnetic beads (STEMCELL™). Up to 97% of the CD11b-positive cells co-expressed either Ly6C or Ly6G (two components of Gr1). (TIF) [file pone.0183271.s003.tif]
